# Role of IgA in the early-life establishment of the gut microbiota and immunity: Implications for constructing a healthy start

**DOI:** 10.1080/19490976.2021.1908101

**Published:** 2021-04-18

**Authors:** Jielong Guo, Chenglong Ren, Xue Han, Weidong Huang, Yilin You, Jicheng Zhan

**Affiliations:** a College of Food Science and Nutritional Engineering, Beijing Key Laboratory of Viticulture and Enology, China Agricultural University, Beijing, China; b Peking University School of Basic Medical Science, Peking University Health Science Centre

**Keywords:** Early life, iga, gut microbiota, immunity, breast milk, proteobacteria

## Abstract

Colonization and maturation of the gut microbiota (GM) during early life is a landmark event that fundamentally influences the (early) immunity and later-life health of various mammals. This is a delicate, systematic process that is biologically actively regulated by infants and their mothers, where (secretory) IgA, an important regulator of microbes found in breast milk and generated actively by infants, may play a key role. By binding to microbes, IgA can inhibit or enhance their colonization, influence their gene expression, and regulate immune responses. IgA dysfunction during early life is associated with disrupted GM maturation and various microbe-related diseases, such as necrotizing enterocolitis and diarrhea, which can also have a lasting effect on GM and host health. This review discusses the process of early GM maturation and its interaction with immunity and the role of IgA (focusing on milk secretory IgA) in regulating this process. The possible application of this knowledge in promoting normal GM maturation processes and immune education has also been highlighted.

## 1. Introduction

Breast milk has long been considered the gold standard for infant food. However, many infants are partially or exclusively formula-fed for various reasons, such as the insufficiency of breast milk or disease prevention (for example, HIV and hepatitis B virus infection).^1,2^ In China’s Sichuang province, ~70% (n = 695) of infants received infant formula as their first food, increasing to 88% within 1 month.^2^ In the USA, only approximately 43% (816 out of 1899) of infants were exclusively fed breast milk at 1 month of age.^3^ Breast milk is significantly more than a nutritional source for infants. It also plays a profound role in shaping the infant gut microbiota (GM) and immune system.^4,5^ Exclusive formula feeding is associated with changes in the GM,^6^ intestinal environment, and incidences of various diseases, such as necrotizing enterocolitis (NEC),^7^ obesity, and allergies.^8^ The primary differences between formula and breast milk include the lack of human milk oligosaccharides (HMOs), microbes, such as *Lactobacillus* and *Bifidobacterium*, and antimicrobial components, such as IgG and IgA in formula.^5,9^

HMOs have received considerable attention over the past few decades. Based on the results obtained from these studies, oligosaccharides, including galacto-oligosaccharides, fructo-oligosaccharides, and polydextrose, have been added to infant formulas to mimic HMOs. These additions close the gap between formula and breastfeeding to some degree, including a decrease in colonic pH and an increase in *Bifidobacterium*.^10^ In addition to oligosaccharides, certain bacteria, or so-called probiotics, mainly members of *Bifidobacterium* and *Lactobacillus* spp., have been used in conjunction with formula, which is believed to benefit infant health. However, the results obtained from these studies are heterogeneous. While some have declared beneficial outcomes from these additions,^11,12^ others have found them to be ineffective or even deleterious.^13,14^ Numerous studies have shown that the efficacy of probiotic supplementation, such as in disease prevention (for example, NEC)^15,16^ and GM restoration,^11^ can only be fulfilled in breast-feeding infants (i.e., in a breast feeding-dependent manner), indicating that certain ingredients in breast milk enhance the function of probiotics. Candidate ingredients include HMOs and secretory IgA (SIgA). However, *Lactobacillus*, the commonly used probiotic for infants,^11^ cannot utilize HMOs. On the contrary, it has recently been discovered that SIgA determines the mucosal colonization of *Lactobacillus*,^17^ suggesting a potential role of SIgA in regulating commensal colonization.

Indeed, gut SIgA has been characterized as a principal regulator of GM (specified below in Section 3).^18^ IgA dysfunction can result in significant gut dysbiosis, including the expansion of (potential) pathogens and increased microbial encroachment on intestinal epithelial cells (IECs), which is implicated in the development of microbe- and inflammation-related diseases (for example, metabolic syndrome (MetS), NEC, and sepsis).^5,19–21^ A balanced interaction between the GM and immune system during early life has a lifelong effect on host health and depends on the regular establishment of GM, a process regulated by (S)IgA in maternal breast milk and the plasma and intestine of infants.^5,22,23^ This specific role of IgA during early life has only been recognized recently. Full identification of properties and functions of IgA during this unique life stage can substantially aid the formation of a healthy GM and immune system in infants.

## 2. Establishment of the GM and its effects on immunity

**Figure 1.**
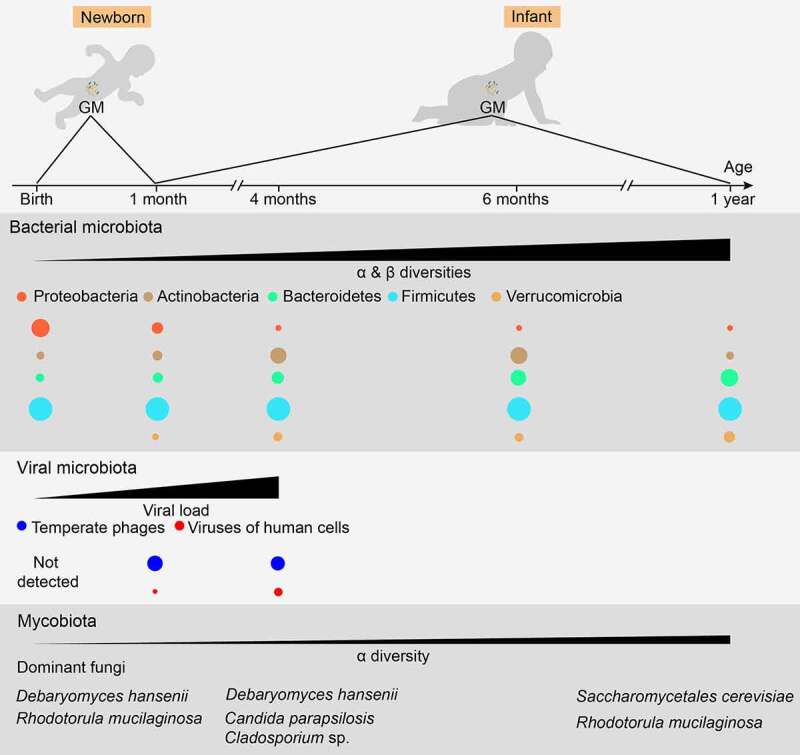
Normal maturation process of early gut microbiota in vaginally delivered, full-term, and breast-fed infants. For bacterial microbiota, the abundance of Proteobacteria is highest during the first few weeks of life, but decreases quickly over time. Actinobacteria, primarily certain *Bifidobacterium* strains, initially increase followed by a decrease, reaching its highest level of richness at around six months of age. The abundance of Bacteroidetes is low at birth but increases steadily during the first year of life. The overall proportion of Firmicutes does not change significantly, showing some fluctuations during maturation. Verrucomicrobia, mainly *Akkermansia muciniphila*, can be detected after one month of age, and its abundance increases over time. For viral microbiota, virus-like particles cannot be detected at birth but their richness increases quickly and reaches 10^9^/g feces at four months of age, a level similar to that in adults. Furthermore, an increase in virus infecting human cells also becomes evident over time. For mycobiota, its α-diversity shows a slight increase over time, while its β-diversity remains mostly unchanged. However, the mycobiota composition changes over time, showing a shift from the early dominance of *Debaryomyces hanseni* to *Saccharomyces cerevisiae* at one year of age. Moreover, changing GM patterns can vary substantially among individuals and can be disrupted by various factors. Therefore, a precise time frame regarding the maturation of early GM compositions needs to be established

The microbiota is found in various body parts, such as the skin, lungs, vagina, and oral cavity. However, microbiota in the gut exhibits the highest microbial loads with a crucial effect on the immunity and metabolism of the host and has received considerable attention over the past few years. Although some studies have demonstrated the existence of bacterial DNA and viable bacteria in the meconium,^24^ placenta,^25^ amniotic fluid,^26^ and fetal intestines^27^ in humans, it is still believed that healthy, vaginally delivered infants obtain their first primary GM during birth from the fecal and vaginal material of their mothers.^28,29^ The bacterial microbiota, virome, and mycobiome of infant GM are significantly different from those of adult GM. These microbial components of infant GM change in delicate processes and mature at different times^6,30,31^ (Figure 1). The correct assembly of these microbial compartments is pivotal for the proper development of early gut immunity, which can have a lifelong effect.^32^

In full-term, breastfed, vaginally delivered healthy infants, the bacterial microbiota is primarily composed of aerobic or facultative anaerobic Proteobacteria and Firmicutes members, such as *Escherichia*/*Shigella* and *Klebsiella, Enterococcus* spp., and *Lactobacillus* during the first weeks of life.^6,33^ The abundance of these bacteria decreases rapidly over the next few months, along with the expansion of the anaerobic bacterial genera, *Bifidobacterium, Clostridium*, and *Bacteroides*. Then, *Bifidobacterium* declines, and adult-like bacterial microbiota is formed at 1–3 years of age. During homeostasis, this adult GM in the colon and feces is dominated by the anaerobic bacteria phyla Bacteroidetes and Firmicutes, accompanied by small proportions of Proteobacteria, Verrucomicrobia, and Actinobacteria.^34^ A higher proportion of Proteobacteria has also been identified in the small intestine than in the colon.^35^

The appropriate interaction between bacterial antigens and the gut immune system during early life imprints immunotolerance to gut commensals and dietary and autologous antigens. The disruption of this process can increase the disposition of immune-related diseases. For example, *Escherichia coli*-derived, but not *B. dorei*-derived, lipopolysaccharides (LPS) can elicit immune responses in primary human peripheral blood mononuclear cells and protect mice from the development of type 1 diabetes.^36^ Finnish and Estonian infants, who have higher levels of *Bacteroides* in their GM during the first 3 years of life than Russian infants, displayed a higher prevalence of early-onset autoimmune diseases.^36^ Similarly, the morbidities of atopy and wheezing at 1 year of age were negatively correlated with the fecal LPS levels at 3 months of age in Canadian infants, although the LPS properties were not characterized.^37^ Interestingly, as discussed above, *Escherichia* forms a significant part of the GM during the first months of life, while *Bacteroides* increases much later. This seems to be a co-evolution between the host and bacterial GM rather than a coincidence.

Bacteriophages belonging to the gut virome, which infect and kill bacterial cells.^38^ The human gut virome is highly dynamic during early life but stabilizes in adulthood.^39^ Virus-like particles are relatively low in the meconium and early feces of healthy infants or cannot be detected by fluorescence staining. Their level increases dramatically and reaches 10^9^/g feces within 1 month, which is similar to that found in adults.^30^ During the first month of life, pioneer bacteria-specific bacteriophages, such as those of *Bifidobacterium* and *Lactobacillus*, dominate the infant gut virome, while the abundance of eukaryotic viruses that infect humans, such as *Adenoviridae, Anelloviridae, Caliciviridae*, and *Picornaviridae*, increased significantly at 4 months of age.^30,38^ In addition, the gut virome can influence the immune responses to bacteria and vice versa. Infecting germ-free (GF) or antibiotic-treated mice with murine norovirus protected them from intestinal injury and pathogenic bacterial infection,^40^ while gram-negative bacteria can induce intestinal antiviral activity in a necrosis factor κB (NF-κB)-dependent manner.^41^

Similar to gut virome, interactions among the bacterial microbiota, mycobiome, and gut immunity have been reported. Clusters IV and XIVa of *Clostridia* resist the colonization of *Candida albicans* via the hypoxia-inducible factor-1α-mediated generation of LL-37 in mice.^42^ The administration of anti-fungal agents exaggerated dextran sulfate sodium (DSS)-induced colitis and house dust mite-induced allergic airway disease, along with bacterial dysbiosis, including a decline in *Bacteroides* and *Clostridium* and an increase in *Streptococcus*.^43^ Unlike bacterial microbiota, fungal diversity changes moderately over time, with a slight increase in alpha-diversity while beta-diversity remains virtually unchanged.^44^ A transformation from *Debaryomyces hansenii* to *Saccharomyces cerevisiae* was evident in *Saccharomycetales* during the first year of life.^44^ Balanced mycobiota in adults mainly include *Candida, Malassezia*, and *Saccharomyces*.^45^

Overall, the establishment of GM during early life is a dynamic process in which bacterial microbiota, mycobiota, viral microbiota, and the gut immune system interact with each other to shape the early gut. This process plays a crucial role in life-long health but can be disturbed by various modern medical practices, such as antibiotics and formula-feeding.^46^ Furthermore, the competition between GM is relatively weak, while the plasticity is strong during this time, making it more practicable to actively build a GM that benefits health.^47^ However, an understanding of the GM maturation process and controlling factors is necessary.

## 3. Role of IgA in the establishment of the GM and immunity

**Figure 2.**
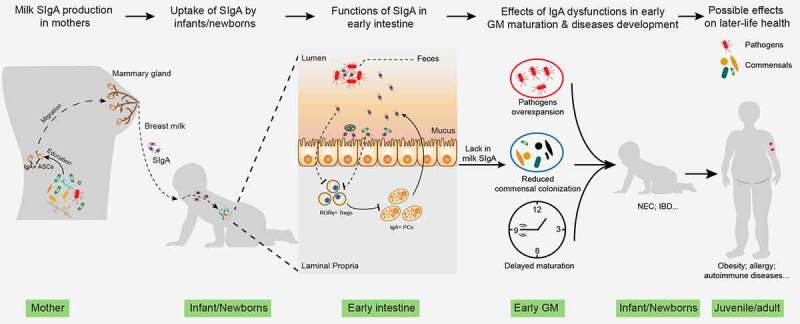
The role of milk SIgA in regulating gut microbiota maturation. IgA+ plasma cells (PCs) produce milk SIgA in the mammary gland, which originates from the gut and is educated by gut microbiota (GM). Milk SIgA is the primary (exclusive) source of intestinal SIgA for breastfeeding infants. SIgA in the intestine can bind to specific pathogens, promoting their clearance via aggregation. The binding of SIgA to certain commensals, such as *Lactobacillus*, can enhance their mucosal colonization. A lack in milk SIgA can lead to the over-enrichment of pathogens and delayed GM maturation, which is associated with the development of various microbe- and immunity-related diseases during infancy (for example, NEC and IBD) and in later life (for example, obesity, allergies, and autoimmune diseases). NEC, necrotizing enterocolitis; IBD, inflammatory bowel disease

Considering the significant effect of an appropriately established GM on health, it is evolutionarily illogical that the host cannot actively regulate this process. Indeed, during pregnancy and lactation, the gut and vaginal microbiota of mothers change in an organized manner, including the expansion of commensals such as *Bifidobacterium* and *Lactobacillus*.^48,49^ These changes are essential for newborns to obtain a healthy initial GM at birth. In addition to programming the first GM, IgA secreted by mothers and infants may also play a role in GM maturation^50^ (Figure 2).

In humans, IgA is the most abundant immunoglobulin isotype secreted into the gut, accounting for three-quarters of the immunoglobulin generated daily at 3–5 g/d.^51^ Naïve B cell precursors expressing IgM and IgD acquire the ability to produce IgA after undergoing IgA class switch recombination (CSR).^52^ Based on the type of antigen and the site of B cell activation, IgA induction has been defined as T cell-dependent (TD) and -independent (TI) pathways. The small intestinal lamina propria (LP) harbors the largest (~80%) IgA+ PC population, while its abundance in colonic LP is small.^35^ Small populations of IgA+ PCs can also be detected in extraintestinal tissues, including the lungs, salivary glands, lactating mammary glands, liver, and bone marrow.^53^ In mucosal tissues, IgA is released together with a fraction of the pIgR, named secretory component (SC), through proteolytic cleavage, resulting in the formation of SIgA.

Gut dysbiosis has been widely reported in mice with IgA-related gene deficiency. RAG-deficient mice cannot generate IgA because of the lack of mature lymphocytes, which exhibit reduced GM diversity and increased intragroup differences in GM composition.^54,55^ Similarly, a reduction in bacterial diversity has been reported in *Ighm*^−/−^ (B cell-deficient) mice compared to that in their wild type (WT) and *Ighm*^+/−^ littermates.^54^ Nonetheless, other immunoglobulin isotypes such as IgM^35^ may also contribute to the observed alteration in GM because of the complete deficiency in B cell function in these two mouse strains. Other studies using IgA- or SIgA-specific-deficient mice further highlighted the important role of (S)IgA in regulating GM composition (specified below in section 3.1.1).^22,23^ Consistent with this, GF mice with a higher abundance and diversity of IgA exhibit a greater ability to diversify the GM after transfer.^56^ Which type of IgA response, TD or TI, dominates GM homeostasis? Contradicting evidences exist on this topic. IgA produced in T cell-deficient mice can bind to a similar group of GM as produced by WT mice,^35^ indicating that TD IgA is dispensable for the maintenance of GM homeostasis. However, mice exhibiting T cell dysfunctions, such as *Cd3e*^−/−^, *Pdcd1*^−/−^, and T-*Myd88*^−/−^ mice, display different GMs compared to WT mice, including a decrease in bacterial diversity and an increase in the abundance of Gammaproteobacteria members *Enterobacteriaceae* and *Desulfovibrionaceae*.^21,54,57^ These results show that although TD and TI IgA bind to a similar group of GM, the outcomes vary owing to different IgA repertoire properties. Consistent with this, mice carrying a knock-in mutation of *Aicda* can still support IgA-generating CSR but not somatic hypermutation (SHM), which shows changes in the composition of their microbiota.^58^

Alteration in the intestinal IgA response to specific bacterial antigens can also change GM composition.^59,60^ Immunization of WT mice with *Salmonella*-derived flagellin increased intestinal flagellin-specific SIgA levels, leading to a decrease in flagellar bacteria, mainly Proteobacteria members.^60^ In contrast, in *Tlr5*^−/-^ mice, there is a lack of immune response to flagellin because of the deficiency in Toll-like receptor 5 (TLR5), a main receptor of flagellin, resulting in an increase in flagellar bacteria.^59^ Collectively, these results demonstrate an important role for IgA in regulating GM.

IgA deficiency is defined as a serum IgA level below minus two standard deviations of the average population-level normalized for age (partial) or as a serum IgA level lower than 0.07 g/L (complete).^61^ In children of all ages, the prevalence of IgA deficiency in healthy individuals, ranging from 1/170 to 1/400, is lower than that in those with recurrent respiratory tract infections, which ranges from 1/4 to 1/65.^61^ Furthermore, 46% of children exhibiting complete IgA deficiency presented with recurrent infections before 1 year of age and 74% before 5 years of age.^62^ These observations indicate a possible role of IgA in controlling microbes during early life. In addition to recurrent infections, children with IgA deficiency are more susceptible to developing other diseases, such as allergic and autoimmune diseases.^63–65^

### 3.1 Breast milk SIgA

Newborns cannot actively generate intestinal SIgA (specified below in the section Intestinal SIgA), rendering the milk SIgA from mothers the exclusive source of intestinal SIgA. IgA is the primary immunoglobulin isotype in breast milk, at a concentration of 15 g/L in colostrum and 1 g/L in mature milk, providing a daily level of 0.5–1.0 g IgA for infants.^66^ Although a small number of antibody-secreting cells (ASCs) can be detected in breast milk, SIgA is mainly produced by PCs in the basolateral region of the mammary glands and secreted into the milk by transcytosis via epithelial cells.^67^ The IgA+ PCs in the mammary gland increase dramatically during pregnancy and lactation and wane after lactation ceases.^50^ They are believed to originate from the intestine mainly because (1) signaling molecules orchestrating the homing of IgA+ PCs to the mammary gland are the same as those in the intestine,^68^ (2) the IgA+ PCs and IgA repertoires in the mammary gland are similar to those in the intestine,^69^ and (3) radioactively labeled intestinal B cells migrate to the mammary gland at a frequency similar to the spleen and bone marrow during pregnancy.^4^

#### 3.1.1 Immune exclusion

An important function of SIgA in the intestine is to enhance the elimination of opportunistic pathogens via agglutination, a function known as immune exclusion^70^ (Figure 2). A recent study has shown that high-avidity intestinal SIgA can bind to the daughter cells of certain intestinal pathogens, such as *Salmonella enterica* subspecies enterica serovar Typhimurium and *E. coli* strains CFT073 and 8178, resulting in incomplete binary fission and the formation of clumps. This enhanced growth is effective at all realistic pathogen densities, promoting the elimination of low-density pathogens in the gut.^71^ Although this mechanism was not verified for *Clostridium difficile*, infants aged between 2.9 and 5.3 months with higher fecal SIgA levels show lower colonization of *C. difficile.^72^*

Proteobacteria accounts for a significant proportion of IgA+ bacteria in fecal and intestinal samples.^18^ Similarly, in humans, Proteobacteria is abundant during the first days of life in mice but decreases rapidly.^22^ Despite being influenced by the gut oxygen content, this process is also controlled by SIgA in the gut. Persistent colonization of Gammaproteobacteria was evident in IgA-deficient (*Igha*^−/−^) mice, resulting in higher susceptibility to intestinal inflammation in neonatal and developed gut.^22^ Notably, although not confirmed by the study, this IgA-mediated inhibition of Gammaproteobacteria could be attributed to SIgA in the milk since young mice cannot actively generate intestinal SIgA until weaning.^23^ Another study using milk SIgA-deficient (*Pigr*^−/−^) mice further demonstrated the pivotal role of milk SIgA in shaping early and adult GMs.^23^ Rogiera et al. found that the GM of both weanling (21 days) and adult (70 days) mice that received no maternal SIgA from their nursing mothers during lactation were different from those of mice that did, including an expansion of *Pasteurellaceae*, belonging to Gammaproteobacteria.^23^ Similar to the results obtained for *Igha*^−/−^ mice, deficiency in maternal SIgA also increased the susceptibility to chemically induced colonic injury in adulthood.^23^

#### 3.1.2 Disease prevention

Many common intestinal (potential) pathogens, such as *Salmonella, Klebsiella*, and *Escherichia*, are Gammaproteobacteria members. Persistent high colonization of Proteobacteria members is a typical characteristic of early gut dysbiosis and is associated with various diseases, such as late-onset sepsis (LOS) and NEC.^73–75^

NEC is an inflammatory mucosal disease with high mortality that affects a significant proportion of preterm infants with a gestational age of less than 33 weeks. The pathogenesis of NEC has not been well defined, but gut dysbiosis, mainly the overexpansion of Gammaproteobacteria, is usually observed before the onset of NEC and is believed to be involved in its development.^74^ As a main controller of the gut Proteobacteria members,^22,23^ SIgA may play a key role in controlling the development of NEC in preterm infants. Indeed, a recent study investigated the role of milk SIgA in the development of NEC and found a decrease in IgA-bound bacteria and an increase in the Gammaproteobacteria member *Enterobacteriaceae* in the IgA-unbound fraction of infants who developed NEC.^5^ This indicated an impaired ability of IgA to bind to Gammaproteobacteria in these infants. *Igha*^−/−^ mice that received no passive SIgA during suckling exhibited NEC development rates that were similar to those of formula-fed mice, while the incidence of NEC in WT control mice was lower, further demonstrating the crucial role of IgA in NEC development.^5^ In accordance, the NEC incidence was substantially lower in breastfed infants than in those who were formula-fed.^76^ Similarly, prenatal stress reduced the IgA levels in mice, resulting in GM alteration in 2-week-old neonatal mice while increasing their susceptibility to experimental NEC.^77^

Meanwhile, it also should be noticed that other mechanism(s) may be also involved in the regulation of disease development by milk SIgA, for example, through the direct regulation of gut immunity (specified below in Section 3.1.5).

#### 3.1.3 Enhancement of mucosal colonization

Besides pathogens, IgA can bind to various commensals, such as lactobacilli, some Clostridia species, and *Prevotella*.^78^ However, as mentioned above, IgA binding can lead to different outcomes because of the discrepancy in IgA properties. In fact, contrary to enhancing the clearance of pathogens by binding to them, IgA binding promotes mucosal colonization and adhesion to the IECs of certain commensals such as *Lactobacillus* and *Bifidobacterium*,^17,79^ which may be crucial for the early colonization of commensals and GM maturation. IgA-coated *Lactobacillus* can colonize the mucous in a glycan-dependent manner.^17^ Disassociation from SIgA or cleavage of glycan side chains of SIgA using a promiscuous N-glycosidase disables the mucosal colonization of *Lactobacillus*,^17^ highlighting the indispensable role of SIgA in mediating the mucosal colonization of *Lactobacillus*. Similar to *Lactobacillus*, an in vitro study using Caco-2 cells demonstrated that SIgA can enhance the adhesion of *Bifidobacterium* to cultured epithelial cells.^79^ These results are also in line with the above-mentioned observations that the efficiency of probiotics supplementation is dependent on breastfeeding.^11,15,16^ In addition, deep colonization of these commensal bacteria within the mucus with the help of SIgA protects them from the predation of bacteriophages whose titer in the mucous is lower than that in the lumen.^80^

Therefore, this unique niche provided by the intestinal SIgA to certain commensals may affect the maturation of early GM. Indeed, children with malnutrition exhibit an overabundance of pathogens, including *Haemophilus, Campylobacter*, and *Escherichia*/*Shigella*, belonging to Proteobacteria.^81^ This is associated with disordered IgA responses to GM, mainly an increase in IgA recognition of pathogens and a decrease in IgA recognition of commensals such as *Verrucomicrobiaceae* and *Bacteroidaceae*.^82^ This impaired IgA recognition of commensals in children with malnutrition is associated with decreased GM diversity and stunted GM maturation.^83^ Moreover, the children involved in these studies ranged in age from 6 months to over 5 years and were not all breastfed. Therefore, intestinal SIgA actively generated by children is also involved in regulating early GM maturation.

#### 3.1.4 Regulation of viral and fungal colonization

The direct binding of SIgA to viruses in the intestine has not been widely documented despite the vaccine-elicited IgA response to specific viruses, such as rotavirus.^84^ Therefore, the direct and overall effects of intestinal SIgA from milk or actively generated by the infants on early viral colonization have not been determined and may be minimal. However, milk SIgA may indirectly affect early viral colonization by regulating the bacterial microbiota in the intestine.^30^ As mentioned above, SIgA can enhance the mucosal colonization of *Lactobacillus*, a common commensal significantly abundant in the early gut and milk.^17^ Similarly, the richness of *Bifidobacterium*, another abundant commensal in the early gut, is positively associated with fecal SIgA levels in young children (from birth to 18 months of age).^85^ Consistent with this, 4-month-old breast-fed infants show an increase in the temperate bacteriophages of *Lactobacillus* and *Bifidobacterium*, which is accompanied with a decrease in viruses that can infect human cells compared to exclusively formula-fed infants.^30^ Moreover, besides SIgA, some other ingredients present in the breast milk, such as oligosaccharides, bacteria, and IgG, can regulate viral colonization^9,86^ therefore, the role of these ingredients in regulating gut virome in breast-feeding infants cannot be ruled out.

On the one hand, the oral colonization of commensal *C. albicans* in mice induced the generation of cross-specific SIgA that binds to both the bacteria and fungi in saliva.^87^ The binding of pathogenic *C. albicans* strain SC5314, by this commensal-induced SIgA prevented their adhesion to and invasion of oral epithelial cells.^87^ Similarly, *S. boulardii* can induce the generation of SIgA against *C. difficile* toxin A in the intestine of mice.^88^ On the other hand, bacterial colonization can also induce the production of antifungal SIgA. One month of *L. casei* and *B. breve* consumption caused an increase in the salivary anti-Candida SIgA levels in elderly humans, accompanied with a slight but significant reduction in the fungal load.^89^ Consequently, SIgA may also mediate the interaction between bacteria and fungi in the mucosa. However, studies investigating the influence of SIgA in the intestine on the overall gut microbiota in adults and its succession during early life, as well as the effect on disease development, are exceedingly rare. This merits further determination since mycobiota are involved in the development of various diseases (for example, Crohn’s disease, reviewed in detail in Ref.^90^) and can interact closely with IgA.

#### 3.1.5 The effect on immune education

By inhibiting the colonization of (potential) pathogens, such as Gammaproteobacteria members and *C. difficile*, the intestinal SIgA from milk may contribute to the management of inflammatory responses to these bacteria and imprint anti-inflammatory properties in the gut immune system.^91^ In addition, the binding of microbial antigens by SIgA can enhance the recognition of these antigens through microfold cells on the PPs and improve the immune responses to these antigens.^56^ The clearance of pathogens, colonization, and interaction with certain commensals (partially) mediated by SIgA during early life can enhance the continued generation of Tregs.^92,93^ Furthermore, early life infection or a lack of interaction with commensals are associated with a reduction in Tregs and increased susceptibility to inflammation and colitis in later life.^92,94,95^

These mechanisms indicate that intestinal SIgA is associated with the generation of Tregs and immune tolerance during early life.^92,94,95^ However, a recent study showed that the levels of colonic RORγ+ Tregs are set up in early life and are negatively correlated with milk SIgA levels ^4^. C57BL/6 J (B6) mice displayed a significantly higher proportion of colonic RORγ+ Tregs than BALB/c mice. This depends on their nursing genotype rather than birth mothers, since mice born to B6 dams but nursed by BALB/c mice show similar levels of colonic RORγ+ Treg than BALB/c mice, and vice versa. This nursing-mediated alteration of RORγ+ Treg is early life- and GM- dependent and is stable to various disruptions.^4^

The abundance of IgA+ PCs in the colon and small intestine was higher in adult BALB/c mice than in B6 mice, while the same applies to fecal and serum IgA levels. Although no significant difference was apparent between the mammary IgA+ PCs of the two genotypes, the IgA levels in breast milk were higher in BALB/c mice.^4^ Pups nursed by BALB/c mice, independent of their birth mothers, show higher fecal IgA levels and IgA+ bacterial proportions, which can also be transmitted through generations.^4^ Furthermore, the colonic RORγ+ Treg level in mice nursed by *Igha*^−/−^ BALB/c mice was higher than that in mice nursed by WT BALB/c mice, independent of their birth mother. A deficiency in RORγ+ Treg leads to increased IgA+ bacterial levels in feces.^4^

These results indicate a double-negative feedback loop between IgA and colonic RORγ+ Tregs. However, Treg is generally thought to be a positive regulator of IgA generation.^18^ Then, why do high colonic RORγ+ Tregs reduce IgA production^4^? Mice with Treg cell-specific c-Maf deficiency (*Maf*^ΔTreg^) display a decrease in IL-10 production but an increase in serum and fecal IgA, as well as substantially elevated fecal IgA+ bacterial levels. Similar results have been obtained from *Il-10*^ΔTreg^ mice, suggesting that IL-10 mediates the Treg-induced inhibition of IgA production.^96^ However, IL-10 is essential for the generation of the IgA CSR.^18^ How does a reduction in IL-10 enhance IgA responses^96^? A possible explanation is that a decrease in IL-10 enhances the gut immune response to GM, leading to an increase in other factors related to IgA generation, such as IL-6, resulting in increased IgA production.^70^ Although it does not generate inflammatory factors, such as NF-κB and TNF-α, the IgA response per se is a unique inflammatory response (type 3 inflammation, pathogen clearance) to microbes.^97^ Consistently, the colonization of microbes in GF mice that generate low-level intestinal SIgA leads to a rapid expansion of the intestinal IgA+ PCs and an increase in IgA production.^98^ *Maf*^ΔTreg^ mice also exhibited high levels of intestinal IL-17A–IL-22-producing TH17 cells, demonstrating an enhanced immune response to GM.^96^

How can IgA continuously influence the RORγ+ Treg level in the colon? Possible mechanisms include the following: (1) IgA binding prevents the encroachment of GM. (2) IgA binding decreases the expression of virulent GM genes. (3) IgA enhances the clearance of GM and inhibits the translocation of their metabolites.^99^ All these mechanisms inhibit the interaction between GM and the host, reducing the immune responses of the host to GM. However, this is inconsistent with observations that IgA-binding promotes the recognition of noninvasive *S*. Typhimurium and *E. coli* and the generation of antigen-specific CD4+ cells and IgA,^56^ which may be attributed to the different life stages of the mice used in these studies.

An increase in the RORγ+ Treg levels in B6-fostered mice induced by low milk SIgA results in increased intestinal injury and bacterial burdens in the gut when challenged by the mouse pathogen *Citrobacter rodentium*.^4^ Furthermore, high RORγ+ Treg proportions are associated with lower susceptibility to colitis, cancer, and allergies, suggesting the importance of a balanced trait.^4^

In summary, as the primary source of SIgA in the early gut, milk SIgA is essential in regulating GM maturation and immune education. Abnormal acquisition of SIgA from milk leads to changes in the patterns of the bacterial, viral, and fungal successions, which is associated with the development of various microbe- and immunity-related diseases.^5,22,23^ Milk SIgA can also directly affect the development of gut immune compartments, primarily colonic RORγ+ Tregs, influencing the immune responses to commensals and disease disposition in later life.^4^ Consequently, a lack of SIgA during early life could be a primary reason for the increased susceptibility to the development of various diseases, including obesity, inflammatory bowel disease (IBD), and NEC, as well as allergic and autoimmune diseases, in exclusively formula-fed infants.^5,100–102^

### 3.2 Intestinal SIgA

The intestinal IgA+ PCs in human infants can only be readily detected after ~1 month of age, the abundance of which increases steadily with time, approaching (but do not reach) adult gut levels at 2 years of age.^103^ Consistently, IgA+ bacteria in the feces of exclusively formula-fed infants was evident until ~30 days of age.^5^ As in humans, mice begin to actively generate intestinal SIgA after weaning (at approximately 21 days of age). Young mice lacking maternal antibodies from the milk generate active intestinal SIgA in advance.^23,104^ Early GM significantly shapes the development of intestinal B cells and has an extended impact on the intestinal SIgA repertoire, which, unlike milk SIgA, can continuously affect intestinal homeostasis.^105,106^

In monocolonized GF mice, increased bacterial exposure causes a decrease in the diversity of intestinal SIgA repertoire.^105^ This corresponds with the observation that intestinal IgA response to abundant commensals leads to the generation of monoclonal IgA in the intestinal SIgA repertoire.^69^ The encountering order also affects the intestinal IgA response to microbes. Colonization of a second bacterium, *C. orbiscindens*, dampens the gut IgA response to the originally colonized bacteria, *E. coli* strain HA107,^105^ promoting the reaction to newly encountered (potential) pathogens.^18^ Moreover, the gut IgA response to specific microbial antigens, such as flagellin, can be long-lasting after transient exposure,^60,107^ which is especially significant in early life.^106^

Dysregulation of the active intestinal IgA response in the early gut is correlated with the development of allergic diseases.^108^ Dzidic et al. investigated of the correlation between the SIgA binding of the GM at 12 months of age, when the generation of active gut SIgA is fairly abundant,^103^ and the development of allergic manifestations at 7 years of age.^108^ They found that children with allergic and asthmatic symptoms showed an altered SIgA recognition pattern of gut bacteria, including a substantial loss of SIgA binding to *Escherichia*/*Shigella*, at 12 months of age.^108^

The maturation of active intestinal SIgA generation in infants (young children) is relatively slow but can be accelerated by weaning. Therefore, it seems that intestinal SIgA acts as a slowly starting relay of milk SIgA during early life, which could be influenced by milk SIgA.^22,23^

### 3.3 Serum IgA

Contrary to milk and intestinal SIgA, serum IgA actively generated by newborns can be detected in small amounts at birth. Most serum IgA is polymeric during infancy^109,110^ and is produced by IgA+ B cells derived from bone marrow. However, in vitro studies showed that serum IgA could bind to a GM group, including Proteobacteria, similar to milk and intestinal SIgA,^111,112^ indicating a correlation between bone marrow- and intestinally derived IgA+ PCs. Indeed, during homeostasis, the generation of serum IgA and bone marrow IgA+ PCs can be significantly enhanced by the enrichment of gut Proteobacteria.^111^ Directly monitoring intestinal B cell migration by photoconverting intestinal tissues also demonstrated a considerable transfer of intestinal IgA+ PCs to the bone marrow.^4^ This GM-boosted serum IgA production protects mice from polymicrobial sepsis during intestinal injury.^111^ Similarly, the abundance of *Clostridium* cluster XI and Proteobacteria is positively correlated with the generation of serum IgA against rotavirus in 6-week-old infants after vaccine treatment.^113^ Therefore, although serum IgA may not directly influence GM maturation, it plays a pivotal role in protecting against gut-derived infections. Based on these observations, serum IgA may complement milk and intestinal SIgA to protect against gut-derived antigens.

### 3.4 Role of IgG and IgM in milk

Breast milk contains considerable amounts of IgG (~0.05 g/L) and (S)IgM (~0.014 g/L),^114^ although at lower levels than (S)IgA, which can influence both GM and immunity in the infant gut.^9, 104,115^ Infecting or intraperitoneally immunizing dams twice with heat-inactivated mouse pathogen, *C. rodentium*, 2 weeks before mating confers protection to their offspring against oral *C. rodentium* challenge at 18 days of age in milk IgG, but not in an IgA- and IgM-dependent manner. This is associated with the virulence recognition factors encoded within the locus of enterocyte effacement pathogenicity island via IgG.^115^ In addition to this pathogen-induced antigen-specific IgG, natural (polyreactive) IgG generated in specific pathogen-free dams or dams colonized with specific *Enterobacteriaceae* members can protect their offspring against the oral challenge of enterotoxigenic *E. coli* at 6–7 days of age.^9^ These results indicate a significant similarity between IgG and IgA. Both the antigen-specific IgA and IgG responses can be elicited by certain pathogens, while IgA and IgG with polyreactivity (especially against Proteobacteria members) are induced by some commensals.^9,112,115^ Moreover, similar to milk (S)IgA, which inhibits the generation of RORγ+ Tregs, milk IgG coordinated with IgA can dampen T cell responses in the neonatal gut in a TLR-dependent but Tcell-independent manner ^104^. However, regardless of these similarities, IgG binds to a smaller fraction and different set of bacteria than IgA in young children.^85^

In contrast, IgM and IgA bind an exceedingly similar subset (mainly *Bifidobacterium* and *Enterobacteriaceae* members) of GM in young children.^85^ Considering the low levels of gut (S)IgM, it may supplement (S)IgA. Indeed, IgA deficiency usually leads to a substantial increase in the intestinal IgM+ ASC and IgM levels in humans.^116^ Only those with compensatory IgM secretion show a normal GM compared to IgA-sufficient individuals.^117^ In summary, it appears that the most abundant antibody in breast milk, SIgA, plays a fundamental role in regulating GM and immunity in the neonatal gut. Furthermore, IgG is crucial for preventing specific pathogens, while (S)IgM is more complementary to (S)IgA.^85,116,117^

## 4. Possible applications for early-life GM regulation via IgA

### 4.1 Demands for the enhancement of early-life IgA functionality

In which situations is it necessary and possible to enhance the IgA functionality to ensure a balanced maturation of the GM and immunity? Exclusively formula-fed infants may be considered first because they do not receive passive IgA through breastfeeding. Second, as the most prevalent primary immunodeficiency, IgA deficiency in children is implicated in the development of various diseases, primarily recurrent infections, as well as allegoric and autoimmune diseases, where impaired regulation of the GM by SIgA during early life may play an important role.^61^ Third, other factors, including antibiotic exposure, birth mode, gestational age, and hospitalization, can disturb early GM, where the supplementation of additional SIgA may help to decrease the effect of these factors on normal GM establishment.

Prolonged antibiotic therapy (≥ 5 days) is usually employed to prevent group B *Streptococcus-*induced early-onset sepsis, while it is associated with the development of LOS.^118^ Infections caused by Gammaproteobacteria members *E. coli* and *K. pneumonia, Staphylococcus* spp., and fungi, such as *Candida* spp., which is commonly seen after antibiotic administration, is implicated in the development of LOS in preterm infants.^75,119,120^ Therefore, antibiotic-induced dysbiosis may contribute to the development of LOS. Since SIgA in the gut is closely related to the clearance of Gammaproteobacteria,^22^ the enhancement of IgA function may contribute to the management of LOS. In addition to LOS, as discussed above, preterm infants are at risk of developing NEC, a deadly disease associated with exclusive formula-feeding and deficient IgA response, which may be prevented by enhancing IgA function.^5^

Although infants are more tolerant to the colonization of *C. difficile*, possibly because they lack the receptors for *C. difficile* toxins during infancy, it can cause diarrhea and increase susceptibility to *C. difficile* infection in adulthood.^121^ Prolonged stay in a neonatal intensive care unit is associated with an increase in the colonization of *C. difficile* in preterm infants,^122^ while a higher fecal SIgA level and breastfeeding are negatively associated with its colonization,^72,121^ indicating the potential role of SIgA in preventing *C. difficile* colonization.

The main difference in the GM of infants delivered via C-section is a decrease in *Bifidobacterium, Lactobacillus*, and bacterial diversity. Supplementation with mixed probiotics, including *B. breve* Bb99, *Propionibacterium freudenreichii* subsp. hermanii JS, *L. rhamnosus* Lc705, and *Lactobacillus rhamnosus* GG (LGG) restores C-section-induced dysbiosis, including an increase in *Bifidobacterium* and a reduction in Proteobacteria in breastfed but not formula-fed 3-month-old infants.^11^ Investigation on the effect of probiotic supplementation on NEC using multiple strains of *Lactobacillus, Bifidobacterium*, and *Streptococcus* indicated a breastfeeding-dependent decrease in the morbidity and mortality of NEC.^15,16^ Similar to probiotics, the effect of fecal microbiota transplantation in restoring C-section-induced dysbiosis in newborns may also depend on breastfeeding.^123^ Although the effect of other possible ingredients in breast milk cannot be excluded, the milk SIgA-mediated enhancement of the colonization of these probiotics (commensals) could be essential, as discussed above.^17^ Furthermore, it indicates that SIgA supplementation would be beneficial when attempting to restore an imbalanced GM in infants when SIgA from milk is not accessible.

### 4.2 Strategies to enhance intestinal IgA function in early life

**Figure 3.**
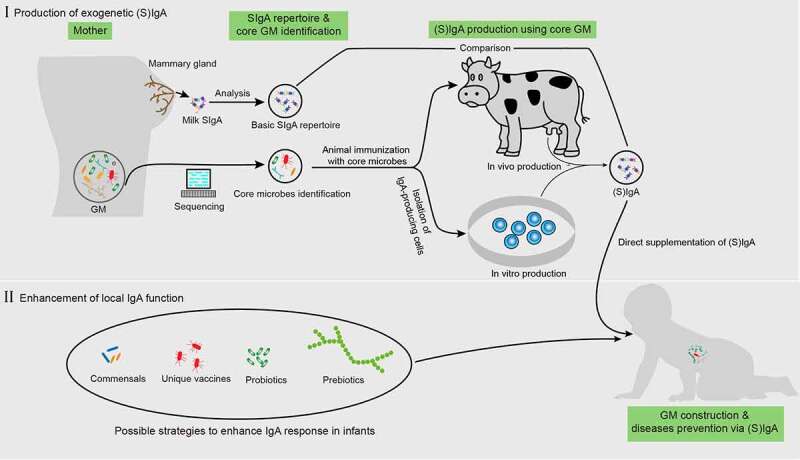
Possible application of IgA to enhance normal gut microbiota maturation. before direct iga supplementation, the “core microbes” must first be identified by analyzing the gut microbiota (GM) properties in pregnant women based on large-scale sequencing. This core microbiota is then used to immunize cows or IgA-producing antibody-secreting cells (ASCs) (or through other possible methods) and obtain IgA. Next, a comparison between the repertoires of this IgA and milk SIgA is also required. For enhancing local IgA functionality, the possible approaches include the supplementation of specific commensals, probiotics, prebiotics, and unique vaccines derived from pathogens

As infants begin to actively generate their own intestinal SIgA after approximately 1 month of age, the capacity to directly enhance local IgA generation is feasible (Figure 3). Probiotics, mainly various *Bifidobacterium* and *Lactobacillus* strain*s*, are well-known for their ability to induce SIgA production.^124^ Supplementation of *Bifidobacterium* and *Lactobacillus* in infants increases fecal SIgA levels while reducing fecal pH and calprotectin, an inflammatory marker.^125,126^ In addition to the direct supplementation of probiotics, infants exclusively fed *the L. paracasei* CBA L74-fermented formula from birth exhibited a significantly higher fecal SIgA level at 3 months of age than those fed a standard formula independent of delivery modes.^127^ Similar results were observed in young children (33 ± 9 months of age), accompanied with a reduction in common infectious diseases in these children.^128^ These studies indicate that probiotic-derived metabolites may be essential for improving SIgA production. Indeed, supplementation of heat-killed probiotics or p40, an LGG-derived protein, promoted SIgA production.^125,129^ This provides a new avenue for enhancing local IgA function that is safer than live probiotics because certain strains of probiotics, such as LGG and *L. acidophilus*, can translocate into the circulation system of patients in intensive care units, contributing to bacteremia, especially in those aged less than 4 years.^13^

In addition to these traditional probiotics, certain commensals reportedly induce intestinal IgA responses in mice and cows.^130,131^ Yang et al. reported that 11 *B. ovatus* strains isolated from humans could strongly induce IgA production in mice.^130^ Notably, this effect does not depend on the colonizing order of these bacteria since the transfer of a multiplex cocktail of these bacterial strains significantly and consistently elevated IgA production in humanized mice.^130^ Moreover, all these bacterial strains were isolated from humans, which are commensals but not (opportunistic) pathogens, suggesting that these bacteria are relatively safe when applied to humans.^130^ Supplementation of the fungus, *S. cerevisiae* boulardii CNCM I-1079, increased the ileal and colonic SIgA and IgA+ PCs in newborn dairy calves at 7 days of age, accompanied with *Lactobacillus* spp. enrichment in the jejunum, corresponding with the ability of IgA to enhance *Lactobacillus* colonization.^17,131^

Dietary fiber-derived short-chain fatty acids can enhance IgA generation in mice by promoting energy production and mRNA expression of genes related to B cell differentiation.^132^ Similarly, 26 weeks of oligosaccharide supplementation in infants from birth resulted in a significant increase in fecal SIgA independent of feeding modes.^133^ As mentioned above, vitamin A (VA)-derived RA is essential for IgA CSR, while VA deficiency leads to a lack of IgA-secreting cells in the small intestines of mice.^134^ In addition, an altered GM with decreased bacterial diversity and an increase in the abundance of Proteobacteria members in mice and children were evident.^135,136^ On the contrary, dietary VA supplementation in 0.5–3.0-year-old children elevated serum IgA levels, decreased diarrhea, and respiratory disease incidences during treatment.^137^

In addition to enhancing the overall IgA response, as mentioned above, it is feasible to strengthen the IgA response against specific microbial antigens. Several immunizations with flagellin derived from Proteobacteria and *Salmonella* induced a persistent IgA response in mice, including increased serum and intestinal (S)IgA.^60^ This flagellin immunization enhanced IECs and GM separation, reduced fecal flagellin levels, and protected mice from diet-induced MetS and chemically induced colitis while decreasing flagellin-producing Proteobacteria, *Desulfovibrionaceae*, in a TD manner.^60^ In contrast, an impaired IgA response to flagellin/Proteobacteria resulted in persistent overexpansion of Proteobacteria members in both newborn and adult mice and an increase in susceptibility to MetS and intestinal injury as discussed above.^20,22^ Therefore, although the safety of flagellin immunization needs further careful determination, this may be a viable approach for controlling the overexpansion of Proteobacteria, a common dysbiosis related to the development of various diseases (for example, NEC, sepsis, malnutrition, and pediatric Crohn’s disease)^5,75,82,138^ in infants.

### 4.3 Direct supplementation with IgA

The strength of the active IgA repose in infants can be impracticable in some cases, such as in children with IgA-related gene deficiency or those less than 1 month of age. Therefore, direct supplementation of IgA in infants may be a better choice (Figure 3).

The main purpose of IgA supplementation in infants is to ensure the normal maturation of their GM. Therefore, two key questions require answers: (1) What is a normal GM maturation process? (2) What is a normal IgA repertoire (i.e., the IgA repertoire that supports normal GM maturation)? As mentioned above, the maturation of early GM can be completed as a process from simple to complex composition and from aerobic/facultative anaerobic to anaerobic bacteria.^18^ However, until now, a precise and overall determination of the composition-time changing schedule of normal GM maturation process, if it exists, has not been well elucidated at the human species level. The determination of this schedule is essential to guide the orchestration of this maturation process. Not only the amount but also the properties of IgA provided to infants affect GM maturation. As discussed above, mice carrying a knock-in mutation of *Aicda* exhibit a defect in SHM, but the IgA-generating CSR is not affected, which is accompanied with altered GM composition.^58^ In addition, a high-fat diet induces changes in IgA repertoire in the spleen and intestine, including an increase in the unmutated IgA proportion, in mice.^139^ Although milk SIgA was not evaluated, considering the gut origin of mammary IgA+ PCs,^4^ it is reasonable to expect a change in the milk SIgA repertoire. Obese mothers display normal milk SIgA levels,^140^ and the bacterial composition in the first neonatal stool (after meconium) of their infants delivered via C-section is also similar to that of infants born to mothers with normal body weight.^141^ However, following the maturation of the GM, young children (about half and most of which were delivered via C-section and are breastfeeding, respectively) born to mothers with obesity showed a different GM composition and exhibited increased susceptibility to obesity compared to those born to mothers with normal body weight,^142,143^ which may be partially attributed to the differences in milk SIgA properties. Therefore, characterizing a basic IgA repertoire that supports normal GM maturation and a core microbial composition capable of inducing this basic IgA repertoire is essential for possible future applications.

How can this basic IgA repertoire and core microbial composition be identified? Direct investigation of the common properties of the IgA repertoire in human breast milk based on large-scale IgA sequencing seems to be a possible approach. However, the IgA repertoire varies significantly among individuals,^144^ making it challenging to summarize commonalities, especially in populations or even at the species level. In addition, during homeostasis, polyreactivity represents the primary selective pressure during B cell maturation in the germinal centers, leading to the binding of a similar group of microbes among individuals.^82^ These phenomena indicate that there is possibly no need to precisely determine the sequencing properties of the basic IgA repertoire. Instead, identifying the core microbial composition that shapes this basic IgA repertoire seems to be more practicable ^105^. During the late stage of pregnancy changes, the GM of women changes regularly, including an increase in Proteobacteria and Actinobacteria members, such as *Bifidobacteria* and *Enterobacteriaceae*.^48^ Proteobacteria members can induce significant IgA responses,^111^ suggesting a possible essential role of Proteobacteria in regulating the milk SIgA repertoire, which, in conjunction with the role of other GM taxa, warrants further investigation.

Another possible application is to inhibit the colonization and (or) promote the clearance of (potential) pathogens in infants using IgA against specific microbial antigens. Similar to the robust induction of anti-flagellin IgA via flagellin immunization in mice, repeated immunization of dietary cows with formaldehyde-inactivated *C. difficile* and partly disabled toxins A and B derived from *C. difficile* substantially induced an IgA response against *C. difficile*, increasing total milk SIgA and SIgA-against *C. difficile*.^145^ Infants who were fed formula supplemented with immunoglobulins concentrated from the milk of cows immunized with *E. coli* showed a decreased incidence of diarrhea.^146^ Although the potential roles of IgM and IgG cannot be excluded (specified below), *E. coli*-specific SIgA may play a central role,^5,145^ also providing a possible approach for the quantity of basic and specific antigen-targeted IgA produced.

The in vitro production of monoclonal or polyclonal IgA using specific antigen-selected B cells can be an avenue for generating highly pure IgA.^112^ However, IgA generated using this method may require an additional combination of the SC, which is essential for the resistance to gastrointestinal digestion and the anchoring of IgA to mucus.^67^

## 5. Conclusions and perspectives

During the extended process of co-evolution between GM and mammals, the host has developed a GM-dependent pattern of early-life immune education. This pattern is exceedingly stable throughout the evolution process, which is challenged by various modern medical and nutritional technologies such as C-section, antibiotic exposure, and formula feeding.^46^ The disturbance of this pattern may be one reason for the increased incidence of various inflammatory diseases, such as allergies and obesity.^46,147^ As a key regulator of GM, IgA influences immune education indirectly by regulating GM maturation and directly affects the immune response.^4^ Disruption of IgA function, including both passive SIgA from breast milk and the local intestinal IgA response, may be essential in impairing GM maturation and immune education processes. Recent studies have shown that IgA is implicated in GM maturation^22,23^ and the development of certain diseases such as NEC and malnutrition during early life,^5,81,82^ indicating a potential role of IgA in shaping a healthy GM and disease prevention. However, studies regarding the role of IgA in early GM and immunity are still relatively lacking. More studies are warranted to further investigate the underlying mechanism(s) and find more possible implications of IgA in disease development. For example, in addition to NEC, disordered interactions between IgA and GM have been implicated in other diseases, including obesity,^19,60^ IBD,^78^ and sepsis.^111^ Although these results were obtained from adult mice or humans, similar patterns may exist in early life. In addition, milk SIgA has been reported to persistently inhibit the generation of intestinal RORγ+ Tregs in a GM- and early life-dependent manner,^4^ and its influence on the development of diseases, such as IBD, merits further investigation.

In addition, although evidence is relatively scarce,^127,138,145^ direct supplementation of SIgA or enhancement of the local intestinal IgA response may be possible avenues to regulate the early life GM maturation process and prevent gut dysbiosis-related diseases. The primary challenges of IgA supplementation in infants include preparation limitations, safety, practicability, and efficacy. IgA preparation can be achieved either in vivo using cows immunized with specific pathogens or colonized with core microbes^145^ or in vitro using specific antigen-selected ASCs.^112^ Efficacy is the foundation of this study. Although some studies have verified the efficiency of IgA supplementation in disease prevention,^146^ thus far, none have evaluated its effect on GM maturation, which may be one of the central aspects for future application, as discussed above. Although a full investigation of the efficacy and safety of IgA supplementation (including IgA preparation strategies) in shaping early GM and disease prevention is difficult, it is relatively easy to preliminarily verify using IgA (*Igha*^−/-^)- and SIgA (*Pigr*^−/-^)-deficient mice, in these cases, SIgA can be prepared from the feces of WT dams.^148^
